# The Association of Common SNPs and Haplotypes in *CETP* Gene with HDL Cholesterol Levels in Latvian Population

**DOI:** 10.1371/journal.pone.0064191

**Published:** 2013-05-13

**Authors:** Ilze Radovica, Davids Fridmanis, Iveta Vaivade, Liene Nikitina-Zake, Janis Klovins

**Affiliations:** 1 Latvian Biomedical Research and Study Centre, Riga, Latvia; The University of Texas Health Science Center (UTHSCSA), United States of America

## Abstract

The heritability of high-density lipoprotein cholesterol (HDL-C) level is estimated at approximately 50%. Recent genome-wide association studies have identified genes involved in regulation of high-density lipoprotein cholesterol (HDL-C) levels. The precise genetic profile determining heritability of HDL-C however are far from complete and there is substantial room for further characterization of genetic profiles influencing blood lipid levels. Here we report an association study comparing the distribution of 139 SNPs from more than 30 genes between groups that represent extreme ends of HDL-C distribution. We genotyped 704 individuals that were selected from Genome Database of Latvian Population. 10 SNPs from *CETP* gene showed convincing association with low HDL-C levels (rs1800775, rs3764261, rs173539, rs9939224, rs711752, rs708272, rs7203984, rs7205804, rs11076175 and rs9929488) while 34 SNPs from 10 genes were nominally associated (p<0.05) with HDL-C levels. We have also identified haplotypes from *CETP* with distinct effects on determination of HDL-C levels. Our conclusion: So far the SNPs in *CETP* gene are identified as the most common genetic factor influencing HDL-C levels in the representative sample from Latvian population.

## Introduction

An inverse association between circulating HDL-C levels and the risk of CAD has been consistently demonstrated in epidemiological studies [1]–[3]. However the recent studies have shown that genetic factors rising plasma HDL-C do not lower risk of myocardial infarction [4].

The heritability of HDL-C level is estimated at approximately 50% [5]. While the high heritability of circulating lipids are well established and earlier studies including recent Genome Wide Association Studies (GWAS) have exposed the involvement of numerous genes and their respective proteins in lipid metabolism [6]–[11], the extent to which each of these factors affect HDL-C level in the general population are largely unknown. Frequent polymorphisms in *CETP, LPL, LIPC, LIPG, ABCA1* and other genes have been reported to be significantly associated with HDL-C levels in multiple populations [7], [12]–[18]. However, in terms of the clinical classification of patients, these variants provide only marginal improvements to the prediction of dyslipidemia or cardiovascular disease [7]. Population based resequencing studies of HDL-C candidate genes have revealed that both common and rare variants of genes associated with altered lipid levels are concentrated in subjects at the extremes of the HDL-C distribution [19], [20], suggesting that studies utilizing subjects from the extremes of the HDL-C distribution may be more powerful than similarly sized population cohorts [13].

Here we report an association study of HDL-C level with SNPs that were found to be significantly associated with HDL-C levels in previously published GWAS. The goal of the study was to use an extreme ends of HDL-C distribution for case/control study design to estimate the role of 144 SNPs from more than 30 candidate gene loci in determining the HDL-C level in Latvian population.

## Methods

### Subjects

We conducted this research using DNA samples from the Genome Database of Latvian Population (LGDB) (shortly described in [21]). Written informed consent was acquired from all LGDB participants. The study protocol was approved by the Central Medical Ethics Committee of Latvia (Protocols Nr. 2007 A-7 and 01-29.1/25). The study group was selected from the total number of 18,888 LGDB participants that were recruited from 2003 to September 2011. During the first stage of sample selection we filtered out all samples that had missing information on standardized measurement of cholesterol (including total cholesterol, HDL-C and LDL-C) and triglycerides. Similarly samples missing information on body mass index (BMI), gender and age were excluded. In total 1581 samples were identified. We excluded all patients with heart and coronary disease conditions (ICD10: from I20 to I70) and those reporting the lipid lowering therapy, leaving 1345 subjects. The first and the last quartile from HDL-C level dispersion were initially selected as cut-off points in order to define two groups of samples leaving 336 samples from each tail. The additional samples were added equally from both tails due to reasons of rationality in order to correspond to 96 well plate format. Thus the final selection of samples represented 28.3% of tails: 380 samples with the lowest HDL-C levels designated as “cases” and 380 samples with the highest HDL-C levels designated as “controls” (total n = 760).

### SNP selection

Our SNP choice was based on information obtained from the number of candidate gene association and GWA studies [7], [10]–[18], [22]–[30]. SNPs were ranked based on their reported highest P values. 191 SNPs were excluded from the list based on low predicted genotyping success value according to manufactures recommendations (Illumina Assay Designe Tool). We then selected top 133 SNPs from this list resulting in 3.49×10^−7^ as the cut-off P value in order to cover the all SNPs from the previous publications that exceeded recommended genome wide significance level of 5×10^−8^ [31]. In addition 11 tag-SNPs were included from the *GP109A/GPR109B* locus using Haploview software and HapMap data resulting in total number of 144 SNPs representing 32 gene loci. The final number of SNPs corresponds to the requirements of the Custom VeraCode GoldenGate Genotyping platform offering estimation of 144 SNP in a single well of a 96-well microplate as one of the standard options. List of all SNPs and respective genes are shown in **Table S1**.

### Genotyping and quality control

All 144 SNPs were genotyped using Illumina BeadExpress system (Illumina Golden Gate genotyping assay). Genotyping was carried out according to the manufacturer's instructions. To ensure quality control and evaluate the intra-subject concordance rate, 46 duplicate samples were randomly distributed in the genotyping plate and one positive control on each assay plate where used. Concordance rates for all assays were greater than 99% and all genotypes of positive controls matched thus no assay plates were excluded. Primary data analyses were performed using Illumina GenomStudio software. The GeneCall threshold was set to 0.25, the cluster images of signal intensity were reviewed manually for all SNPs. Four SNPs were excluded because it was impossible to carry out their allelic clusterization (rs676823, rs1429974, rs3922628 and rs676404). Samples with call rates lower than 0.950 were excluded (n = 56). Further data analysis was performed using toolset PLINK and as the result one SNP (rs11591147) was excluded since it did not pass a Hardy-Weinberg test (P<0.001). After quality control, the remaining sample consisted of 704 individuals genotyped on 139 SNPs, thus attaining the genotyping rate of 99.83%.

### Statistical analysis

Statistical analysis was carried out using PLINK v2.050 software (http://pngu.mgh.harvard.edu/purcell/plink/) [32]. Logistic regression was used to test the difference between the cases and the controls adjusting for sex, age, BMI, triglycerides and LDL-C. Bonferroni correction was used to calculate the significance level (0.05/139 = 3.5×10^−4^). SNPs in genes with more than one significantly associated SNP were assigned to haploblocks that were generated using genotype data from HapMap1/3 with Haploview software v4.2 [33]. The association analysis of haplotypes was performed by PLINK toolset. Conditional haplotype analysis was performed using PLINK, testing whether each of the associated SNPs have an effect that is independent of the haplotypic effects of other SNPs from the particular haploblock. Haplotype and risk allele dosage calculations were performed using GraphPad InStat (GraphPad Software, San Diego California USA, www.graphpad.com). Statistical power was calculated using Quanto v1.2.4 software (Natara Software, Naperville, Illinois, USA, http://hydra.usc.edu/gxe) [34]. Our sample size provided 80% power (at α = 0.05) to detect an odds ratios from 1.34 to 2.31 depending on MAF of different SNPs.

## Results

**Figure 1:**
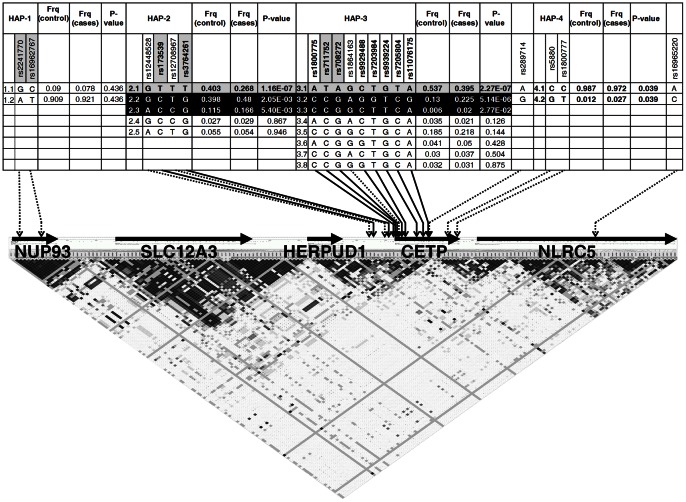
Location of genetic markers in *CETP* gene locus and haplotype association results. The upper part of the figure shows the haplotype association with low HDL-C levels. Haplotypes exceeding 1% frequency are shown. Frequency of each haplotype in cases and controls, and p-value from logistic regression is displayed. Significantly associated SNPs are in bold. Risk haplotypes are black filled, while protective haplotypes are grey filled. Lower part of the figure shows the schematic representation of *CETP* gene and pair wise LD plot. LD plot was calculated using HapMap data. Genes within the locus are indicated by thick, black horizontal arrows. Positions of the genotyped SNPs are displayed by arrows – filled arrows represent significantly associated SNPs and dotted arrows show positions of SNPs not associated with low HDL-C.

In this study we genotyped a set of 144 SNPs in total of 760 DNA samples from LGDB. We selected 380 samples from extreme ends of HDL-C level distribution of 1345 samples. 139 of genotyped SNPs and 704 of included samples (361 controls and 343 cases) passed all quality control procedures and were suitable for further analysis. Detailed characteristics of the study subjects are listed in **Table 1**. Surprisingly, the mean age was identical in cases and controls. In the same time there were significantly more females in the control group than in the case group and the subjects in the control group had significantly lower body mass index (BMI) than the subjects in the case group. Control group had significantly higher total cholesterol, triglyceride and LDL-C levels than case group. We therefore performed a logistic regression analysis including these variables as covariates in addition to an allelic association test. Within the filtered dataset all 139 SNPs were in Hardy Weinberg equilibrium and displayed average genotyping rate of 99.83%. As the result of allelic association we detected 34 SNPs located within 10 candidate genes (*CETP, LCAT, LPL, TOMM40, LIPG, MLXIPL, HMGCR, APOA1, APOA5* and *NIACR1*) with nominally significantly different distribution of alleles between cases and controls (**Table 2**). Most of the association remained at the same significance level after the logistic regression analysis adjusting for gender, age, BMI, triglycerides and LDL-C with exception of 13 SNPs from *CETP*, *HMGCR*, *LPL, APOA1, APOA5* and *MLXIPL* genes. The association data for all SNPs are presented in **Table S1**. The effects for all associated SNPs where in the same direction as in previous studies. After applying the Bonferroni correction 10 SNPs remained significantly associated (p<3.5×10^−4^). All of these 10 SNPs were located in *CETP* gene. In order to evaluate the role of individual SNPs in association with decreased HDL-C levels we also performed a haplotype analysis for *CETP* gene. We thus selected all 19 SNPs from our panel that were located within or in near proximity of *CETP* gene and assigned them in four haploblocks that were generated from the HapMap1/3 data. Two SNPs were located in haploblock, which enclosed *NLP93* gene region (HAP1), four SNPs were located in the haploblock upstream of *CETP* gene (HAP2), nine SNPs were within the haploblock covering the 5′UTR and part of the *CETP* gene (HAP-3), and two SNPs were in the haploblock covering 3′ part of *CETP* and UTR (HAP-4). Rs289714 was located between HAP3 and HAP4 and only one SNP was located in the haploblock covering the *NLRC5* gene that is located downstream of the *CETP*. Location of all 19 SNPs within the haploblocks of *CETP* gene region are shown in **Figure 1**. Acquired haplotype data was further used for haplotype association test. Haplotype reconstruction results indicated the following haplotypes that exceeded frequencies of 0.01: two haplotypes of HAP-1 haploblock, five of HAP-2, eight of HAP-3, and two of HAP-4 haploblocks. One haplotype of both HAP-2 and HAP-3 (HAP-2.1 GTTT P = 1.16×10^−7^; Odds Ratio (OR)  = 1.84 95% Confidence Interval (95% CI)  =  (1.47–2.30) and HAP-3.1 ATAGCTGTA P = 2.27×10^−7^; OR  = 1.78 95% CI  =  (1.44–2.20) respectively) were significantly more frequent in the group with high HDL-C levels and thus were designated as “protective”, while two haplotypes of both HAP-2 and HAP-3 haploblocks (HAP-2.2 GCTG P = 0.002^3^; OR  = 0.72 95% CI  =  (0.58–0.89), HAP-2.3 ACCG P = 0.0054×10^−3^; OR  = 0.65 95% CI  =  (0.48–0.88) and HAP-3.2 CCGAGGTCG P = 5.14×10^−6^; OR = 0.51 95% CI  =  (0.39–0.68), HAP-3.3 CGGCTTCA P = 0.027×10^−2^; OR  = 0.34 95% CI  =  (0.12–0.93) respectively) were significantly more frequent in group with low HDL-C levels and were designated as “risk” haplotypes.

**Table 1:** Sample characteristics.

Variable	High HDL-C level group (controls)	Low HDL-C level group (cases)	P-value
n	361	343	
SNPs genotyped	139	139	
Genotyping rate, %	99.84	99.83	
Mean age, years ± SD (min-max)	52±13 (18–81)	52±14 (18–82)	0.7857
Female gender, %	82.5	43.1	<0.0001
HDL-C level, mmol/L ± SD (min-max)	2.13±0.29 (1.81–3.52)	1.10±0.16 (0.33–1.31)	<0.0001
BMI, kg/m^2^ ± SD (min-max)	24.71±3.99 (15.64–41.91)	29.43±5.34 (16–59.729)	<0.0001
TH level, mmol/L ± SD (min-max)	6.06±1.33 (3.69–14.02)	5.59±1.40 (1.95–13.6)	<0.0001
LDL-C level, mmol/L ± SD (min-max)	3.44±1.13 (1.07–9.14)	3.65±1.18 (0.93–9.66)	<0.0001
TG level, mmol/L ± SD (min-max)	1.06±0.45 (0.34–3.43)	1.95±0.93 (0.41–5.87)	<0.0001

HDL-C – high-density lipoprotein cholesterol; MBI – body mass index; TH – total cholesterol; LDL-C – low-density lipoprotein cholesterol; TG – triglycerides.

**Table 2:** SNPs associated with HDL-C level.

SNP	Corresponding gene	Risk allele	RAF (controls n = 361)	RAF (cases n = 343)	Logistic regression P-value	Logistic regression P-value after adding covariates (gender, age, BMI, triglycerides and LDL-C)	Odds Ratio (95% Confidence interval)
**rs1800775 ** [**7**], [15], [17], [18], [23], [27], [**29**]	**CETP**	**C**	**0.37**	**0.52**	**8.60E–08**	**4.51E–07**	**1.85 (1.50–2.29)**
**rs3764261 ** [**7**], [11], [12], [14], [23], [27****]	**CETP**	**G**	**0.60**	**0.73**	**1.49E–07**	**3.15E–05**	**1.83 (1.46–2.29)**
**rs173539 ** [**23]**, [24****]	**CETP**	**C**	**0.59**	**0.73**	**2.47E–07**	**2.73E–05**	**1.81 (1.45–2.27)**
**rs708272 ** [**18**]	**CETP**	**G**	**0.47**	**0.61**	**3.06E–07**	**9.28E–06**	**1.73 (1.40–2.13)**
**rs711752 ** [**23]**, [25****]	**CETP**	**C**	**0.47**	**0.61**	**3.50E–07**	**1.55E–05**	**1.73 (1.40–2.13)**
**rs9939224 ** [**23**]	**CETP**	**T**	**0.14**	**0.24**	**7.39E–07**	**2.48E–05**	**2.04 (1.55–2.69)**
**rs7205804 ** [**7]**, [23], [25****]	**CETP**	**C**	**0.48**	**0.60**	**3.41E–06**	**1.57E–04**	**1.64 (1.32–2.02)**
**rs7203984 ** [**7]**, [**23**]	**CETP**	**G**	**0.15**	**0.24**	**6.06E–06**	**1.05E–04**	**1.88 (1.44–2.47)**
**rs11076175 ** [**23**]	**CETP**	**G**	**0.13**	**0.22**	**2.08E–05**	**8.04E–05**	**1.87 (1.41–2.48)**
**rs9929488 ** [**23**]	**CETP**	**G**	**0.23**	**0.32**	**6.08E–05**	**7.80E–04**	**1.64 (1.29–2.08)**
rs1864163 [7], [11], [18], [23]	CETP	A	0.21	0.29	0.0007	0.01822	1.54 (1.20–1.96)
rs9891572 [27]		C	0.76	0.83	0.00224	0.15920	1.50 (1.16–1.95)
rs289714 [23]	CETP	G	0.12	0.17	0.00292	0.02975	1.57 (1.16–2.13)
rs12708967 [23]	CETP	C	0.14	0.19	0.00996	0.04379	1.44 (1.08–1.91)
rs2271293 [24]	LCAT	G	0.82	0.87	0.01046	0.01664	1.45 (1.08–1.94)
rs12448528 [7], [23]	CETP	A	0.17	0.22	0.01305	0.05439	1.39 (1.06–1.81)
rs662799 [11], [12]	APOA1	C	0.05	0.08	0.01322	0.20080	1.73 (1.11–2.69)
rs6507945 [17]	LIPG	A	0.42	0.49	0.01392	0.00867	1.30 (1.05–1.6)
rs2410630 [7]	LPL	C	0.57	0.64	0.01395	0.05329	1.30 (1.05–1.61)
rs255049 [27]	LCAT	A	0.77	0.82	0.01536	0.08217	1.37 (1.05–1.77)
rs12679834 [23]	LPL	A	0.93	0.96	0.01745	0.01243	1.76 (1.10–2.80)
rs10096633 [7], [23], [27]	LPL	G	0.91	0.94	0.02088	0.00644	1.63 (1.07–2.47)
rs328 [12], [15], [23], [25], [27], [29]	LPL	G	0.93	0.96	0.02262	0.01283	1.72 (1.08–2.75)
rs2035191 [7]	HMGCR	A	0.79	0.83	0.02754	0.04028	1.35 (1.03–1.77)
rs1551894 [7]	HMGCR	C	0.74	0.79	0.02841	0.17460	1.32 (1.03–1.70)
rs6589566 [10]	APOA5	C	0.05	0.08	0.02854	0.18620	1.60 (1.05–2.45)
rs1919484 [17], [23]	LPL	C	0.72	0.77	0.03068	0.10790	1.30 (1.02–1.65)
rs5880 [18], [25]	CETP	G	0.02	0.04	0.03289	0.32230	2.15 (1.09–4.22)
rs3846662 [7], [12], [22]	HMGCR	T	0.53	0.59	0.03484	0.36730	1.25 (1.01–1.55)
rs405509 [7]	TOMM40	A	0.41	0.46	0.03511	0.02218	1.25 (1.01–1.54)
rs11974409 [7], [12]	MLXIPL	T	0.83	0.87	0.03942	0.06858	1.37 (1.02–1.84)
rs1800777 [18], [25]	CETP	T	0.01	0.03	0.04388	0.02083	2.26 (1.01–5.02)
rs7314976	NIACR1	A	0.19	0.23	0.04408	0.29850	1.31 (1.01–1.70)
rs2000571 [11]	APOA1	A	0.24	0.29	0.04418	0.02341	1.28 (1.01–1.62)

RAF – Risk Allele Frequency; LDL-C – low-density lipoprotein cholesterol; SNPs significantly associated with HDL-C level after Bonferroni corection are in bold.

Conditional halpotype analysis where performed to identify SNPs with independent effects in each haploblock. As a result only the rs9939224 from HAP-3 haploblock displayed effect (P-value  = 0.0275) that was independent from other SNPs.

**Figure 2:**
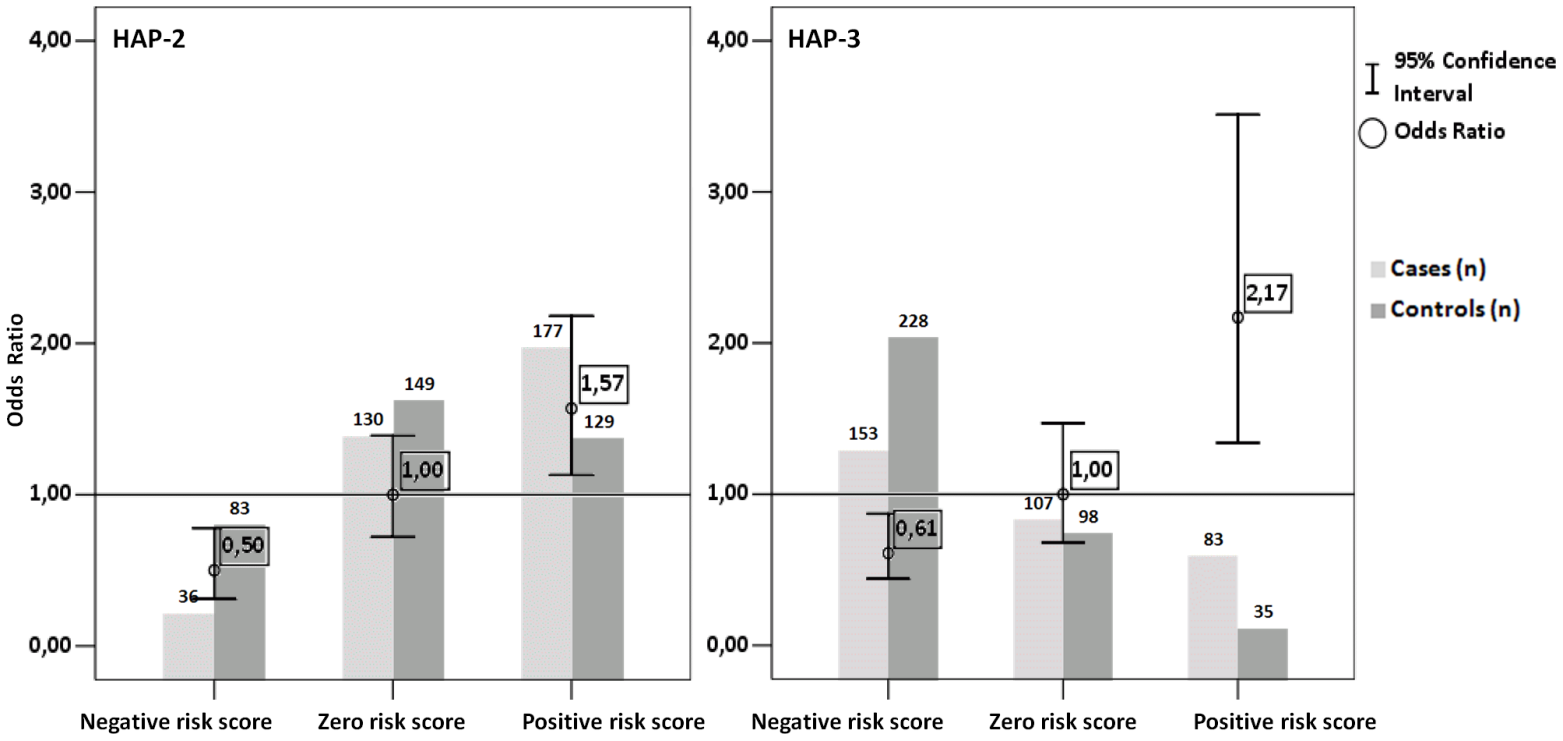
Association of haplotype combination from *CETP* gene with the presence of low HDL-C level. Negative risk score represents individuals with one or two “protective” haplotypes; zero risk score represents individuals with “neutral” haplotypes or one “protective” and one “risk” haplotype, Positive risk score represents individuals with one or two “risk” haplotypes. Subjects with zero risk score were used as reference group. Light grey columns indicate number of individuals with low HDL-C levels; dark grey columns indicate number of individuals with high HDL-C levels. Error bars represent confidence intervals at 95%.

In order to estimate the risk for low HDL-C depending on haplotype combination from haploblocks HAP2 and HAP3 we assigned a value of −1 for each of protective haplotypes (HAP-2.1 and HAP-3.1) and a value of +1 for each of the risk haplotypes (HAP-2.2, HAP-2.3, HAP-3.2 and HAP-3.3). The individuals were then grouped in three groups defined by negative, zero or positive risk scores. We found that individuals with negative score have significantly lower risk (P = 0.0024; OR  = 0.50 95% CI  =  (0.31–0.78)) in case of HAP-2 and (P = 0.0052; OR  = 0.61 95% CI  =  (0.44–0.87)) HAP-3 haploblocks, while individuals with positive score have significantly higher risk (P = 0.0065; OR  = 1.57 95% CI  =  (1.13–2.18)) in case of HAP-2 and (P = 0.0014; OR  = 2.17 95% CI  =  (1.34–3.51)) HAP-3 haploblocks for low HDL-C compared with zero score group. Results of this analysis are shown in **Figure 2**.

**Figure 3:**
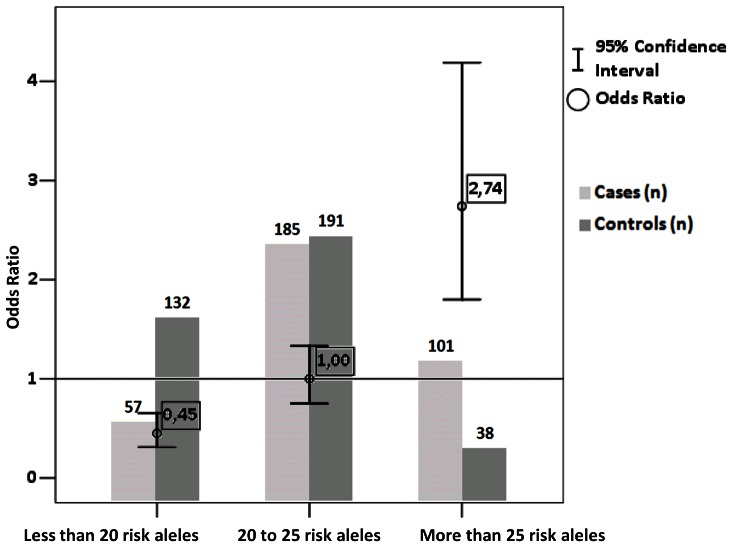
Joint effects of risk alleles on the presence of low HDL-C level. Odds ratios with corresponding 95% confidence interval are shown. Subjects with 20 to 25 risk alleles were used as reference group. Light grey columns indicate number of individuals with low HDL-C levels; dark grey columns indicate number of individuals with high HDL-C levels.

Similarly to investigate the summary effects of risk allele number on low HDL-C level from other genes, we performed risk allele dosage test. For this analysis we choose 20 of 34 SNPs which were nominally associated with low HDL-C level before the Bonferroni correction and each represented one haploblock. In cases with more than one nominally associated SNP in each haploblock we choose one with the lowest P-value. As a result no or low LD (r^2^<0.54) was observed between any pair of these SNPs with exception of rs328 and rs12679834 (r^2^ = 0.99) located in *LPL* gene. In total 20 groups of individuals were obtained with number of risk alleles ranging from 13 to 32 with estimated median of 22.5. We then divided our sample in three equal groups with respect to the median value. First group included those individuals with 13 to 19 risk alleles, second group contained individuals with 20 to 25 risk alleles and the third group represented individuals with 26 to 32 risk alleles. As a result (**Figure 3**) we discovered statistically significant association between risk allele number and HDL-C level. Individuals with less than 20 risk alleles are more frequent in the group with high HDL-C (P = 1.59×10^−5^; OR  = 0.45 95% CI  =  (0.31–0.65)), while individuals with more than 25 risk alleles are more frequent in the low HDL-C group (P = 1.98×10^−6^; OR  = 2.74 95% CI  =  (1.80–4.19)).

Our study was sufficiently powered (80%) to detect OR for SNPs association with decreased HDL-C from 1.85 to 1.4 for SNPs with MAF from 0.059 to 0.27 respectively. For SNPs with MAF >0.27 we would not be able to confirm the absence of association with 80% power for Odds Ratio below 1.4.

## Discussion

This and other studies support the genetic contribution of common variants as determinants of blood lipid phenotypes in the population. We found that in our study group 10 SNPs, located in *CETP* gene (**Table 2**), were the most strongly associated with altered HDL-C level. This finding is in good agreement with the results gained from previous studies [11], [12], [14]–[18], [23], [24], [35], [36], confirming the *CETP* gene defects as the major cause for high HDL-C level. Our results are also in a good agreement with CETP function in lipid metabolism. CETP promotes the transfer of cholesteryl esters from HDL-C to apolipoprotein B (apoB)-containing particles in exchange for triacylglycerols, enabling the receptor mediated uptake of cholesterol esters by the liver and decreasing the HDL-C level [37]. CETP deficiency on the other hand increases the HDL-C level.

The selection of study group was based on HDL-C levels, comparing groups from extreme ends of HDL-C distribution (**Table 1**). Not surprisingly there was a significant difference between gender distributions among groups as the gender-specific genetic differences for lipid traits are well known [38].

The fact that the first three most associated SNPs (rs1800775, rs3764261 and rs173539) of all tested SNPs are located in *CETP* gene promoter region suggests that alterations in CETP expression is the most common reason for subsequent distortion in HDL-C level. These results are again in good agreement with previous studies where the functionality of rs1800775 has been linked to changes in binding site Sp1/Sp3 [39], [40]. Rs3764261 and rs173539 in our study however are more likely associated because of the strong LD with eventually functional SNP rs183130 [36] that was not tested in our study. Since the number of associated SNPs was found in *CETP* gene, we performed more detailed analysis of haplotype distribution between cases and controls in order to evaluate the role of different haplotypes in determination of HDL-C levels. Pair wise LD values and haploblock structure for genotyped SNPs in the CETP locus are shown in **Figure S1**. It should be noted that Haploblock structure derived from genotyped data corresponds very well to the haploblocks derived from HapMap1/3 data. Most of the associated SNPs fall in two haploblocks HAP-2 and HAP-3 (**Figure 1**). HAP-2 is located ∼2 kb upstream *CETP* gene, whereas the HAP-3 haploblock include ∼0,6 kb region upstream *CETP* gene, promoter and first ∼10 kb of *CETP* gene. For HAP-2 it is clearly seen that difference in haplotype distribution between cases and controls is explained by presence of risk alleles from two SNPs rs173539 and rs3764261 that both are in strong LD with each other (R^2^ = 0.94). Conditional haplotype analysis for HAP-3 indicated the independent effect of rs9939224 that is different from haplotypic effects represented by other associated SNPs (rs708272, rs711752, rs7203984, rs7205804 and rs11076175). T allele of Rs9939224 that is located in the second intron of CETP gene is present only in both “risk” haplotypes and one may speculate that this allele may be linked to the increased expression or functionality of CETP, thus decreasing the HDL-C levels. The effect of “protective” haplotype on the other hand is largely explained by the presence of rs1800775 A allele. These results thus would suggest the presence of three (protective, neutral and risk) functionally distinct haplotypes within HAP-3 depending on the combination of alleles from these two SNPs.

In order to test whether the presence of various combinations of “risk” and “protective” haplotypes alters the HDL-C levels, we performed haplotype dosage analysis for both HAP-2 and HAP-3 haploblocks. As the result we found that there is an increase of effect in the presence of one or two “risk” haplotypes in both haploblocks. The observed increase of effect was 1.57 times for HAP-2 in the presence of one or two “risk” haplotypes and 2.17 times for HAP-3 in a presence of one or two “risk” haplotypes (**Figure 2**). Some of the data gained in this study were similar to those acquired by Ridker et al. during his recent study on *CETP* gene association with risk of myocardial infarction [18]. The results of this research suggested that *CETP* gene have an impact on the risk of CAD. However there are some uncertainties whether CETP has pro- or antiatherogenic role [37]. Hirano and coworkers have shown that individuals with reduced CETP function and high HDL-C levels in combination with hepatic lipase activity have increased risk for CAD [41]. Moreover in population study of Japanese men it was shown that males with CETP defects and low or mildly increased HDL-C levels had an increased risk for CAD comparing to those with no CETP defects but with similar HDL-C levels. In contrast men with very high HDL-C levels had decreased risk for CAD regardless CETP defects [42]. This indicates that further studies are needed to investigate the actual role of the influence of *CETP* gene on CAD. Some animals including the mice and rats are naturally CETP deficient and according to discussed antiatherogenic role of this protein rodents are relatively resistant to atherosclerosis. In contrast rabbits do express CETP (like humans) and are susceptible to atherosclerosis [37]. Even though there are strong environmental factors that influence susceptibility to atherosclerosis this suggests that CETP-expressing mammals have increased risk for atherosclerosis [37].

The most probable explanation for the lack of highly significant association with other studied SNPs is the lack of power due to relatively small sample size. This is similar to the previous GWAS, in which the *CETP* was the only gene reaching the genome-wide significance at the initial screening level [23]. Nevertheless we performed further analysis for those SNPs that were nominally associated with HDL-C levels. These 34 SNPs are located in10 genes. Results indicate that increased number of risk alleles (more then 25) is significantly associated with decreased HDL-C level (OR  = 2.74 95% CI  =  (1.80–4.19)), while the presence of less than 20 risk alleles is significantly associated with higher HDL-C level (OR  = 0.45 95% CI  =  (0.31–0.65)) (**Figure 3**). Further case-control studies with CAD patients are needed to test whether risk allele number affects CAD risk as well. Six of these nine genes encode proteins that are directly involved in different lipid metabolisms: APOA1, APOA5, HMGCR, LCAT, LIPG and LPL. APOA1 is one of the major components of HDL-C in plasma, defects in *APOA1* gene are associated with HDL-C deficiencies, including Tangier disease [43], [44]. APOA5 has a role in determining the plasma triglyceride levels in an age-independent manner [45]. HMGCR catalyzes the committed step in cholesterol biosynthesis, but it has other functions not related to lipids as well. Two mutations in this gene are significantly associated with reduced efficacy of pravastatin therapy in reducing cholesterol level [46]. The protein encoded by *LCAT* gene doses the esterification of cholesterol which is required for cholesterol transport in cells, mutations in this gene can cause fish-eye disease as well as LCAT deficiency [47]. LIPG regulates the circulating level of HDL-C and also trough non-enzymatic action can increase cellular lipoprotein uptake and monocyte adhesion and contribute to atherosclerosis [48]. And finally *LPL* encodes lipoprotein lipase which has dual functions acting as triglyceride hydrolase and ligand/bridging factor for receptor-mediated lipoprotein uptake [49]. One of the *LPL* gene polymorphisms that acts in concert with other SNPs in the *PLTP* and *CETP* genes affect plasma levels of HDL-C [50]. The three remaining genes nominally associated with low HDL-C levels (*MLXIPL, NIACR1* and *TOMM40*) do not influence lipid metabolism directly, and further functional studies are needed to investigate the mechanisms through which they influence HDL-C level. We were not able to directly estimate the power of our study, since previously published studies utilized population samples for quantitative trait association while we are comparing two groups representing extreme ends of DHL-C distribution. However due to the relatively small sample size we cannot confirm the absence of association in our study population for SNPs with small effect sizes, which is the main limitation of this study.

### Conclusions

The results gained during this study confirm that from all genes included in the analysis the *CETP* gene is the strongest genetic factor influencing the plasma lipid level in our study group. It should be noted that this is the first replication study considering the population from Baltic States and eastern part of the Europe.

## Supporting Information

Figure S1 **LD plot of genomic locus containing** ***CETP*** **gene.** LD was determined considering all genotypes obtained in our study. Haploblocks identified using Haploview software v4.2 are shown by black lines, but different dotted lines represents haploblock boundaries calculated from HapMap data.
(TIF)

Table S1 **All SNP data.**
(DOCX)
