# Supplementary figures and images for: The Association of Common SNPs and Haplotypes in CETP Gene with HDL Cholesterol Levels in Latvian Population
Source: PLoS One. 2013 May 13;8(5):e64191. doi: 10.1371/journal.pone.0064191 (PMC3652817; doi:10.1371/journal.pone.0064191)

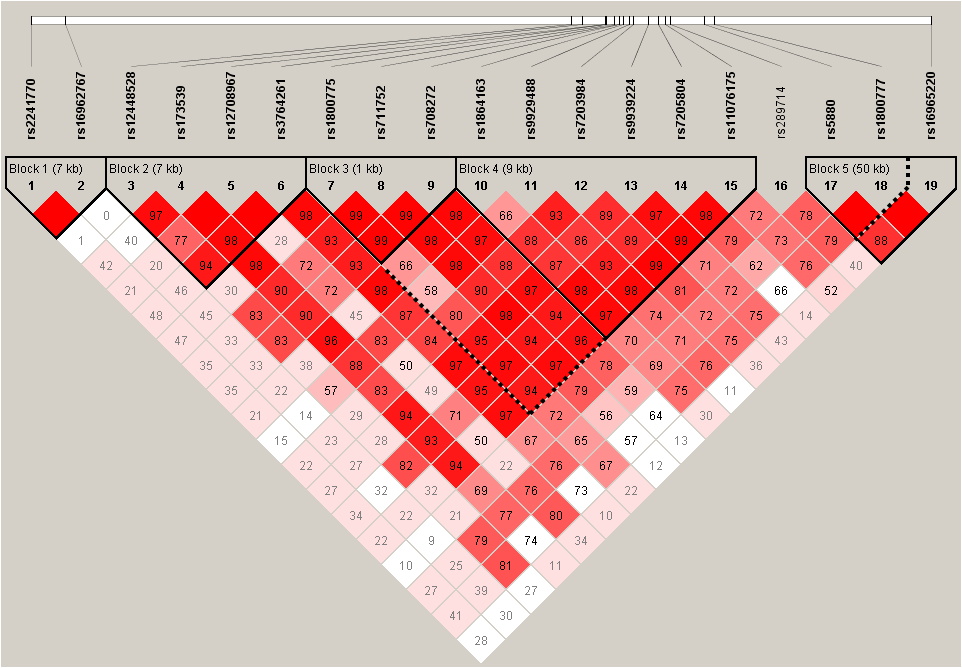

Supplement: Figure S1 — LD plot of genomic locus containing CETP gene. LD was determined considering all genotypes obtained in our study. Haploblocks identified using Haploview software v4.2 are shown by black lines, but different dotted lines represents haploblock boundaries calculated from HapMap data. (TIF) [file pone.0064191.s001.tif]
