# Supplementary material for: The Association of Common SNPs and Haplotypes in CETP Gene with HDL Cholesterol Levels in Latvian Population
Source: PLoS One. 2013 May 13;8(5):e64191. doi: 10.1371/journal.pone.0064191 (PMC3652817; doi:10.1371/journal.pone.0064191)
Supplement: Table S1 — All SNP data. (DOCX) [file pone.0064191.s002.docx]

**Supplemenraty Table 1. All SNP data**

| SNP | Chr | Position | Corresponding gene | Risk allele | MAF | p-value Hardy Weinberg | Genotyping rate, % | Risk allele frequency in group with higher HDL levels (controls n = 361) | Risk allele frequency in group with lowest HDL levels (cases n=343) | P-value | Odds Ratio (95% Confidence interval) |
| --- | --- | --- | --- | --- | --- | --- | --- | --- | --- | --- | --- |
| rs11206510 [12-15] | 1 | 55496039 | PCSK9 | G | 0.134 | 0.146 | 99.86 | 0.13 | 0.14 | 0.66310 | 1.07 (0.79-1.45) |
| rs11591147 [12] | 1 | 55505647 | PCSK9 | T | 0.000 | 1.50E-48 | Excluded |  |  |  |  |
| rs1167998 [12] | 1 | 62931632 | DOCK7 | G | 0.328 | 0.040 | 99.86 | 0.32 | 0.33 | 0.75636 | 1.04 (0.83-1.29) |
| rs10889353 [12, 13] | 1 | 63118196 | ANGPTL3 | A | 0.309 | 0.113 | 100.00 | 0.69 | 0.69 | 0.82296 | 1.03 (0.82-1.29) |
| rs12130333 [12-14] | 1 | 63191777 | ANGPTL3 | C | 0.192 | 0.904 | 100.00 | 0.80 | 0.81 | 0.58523 | 1.08 (0.83-1.40) |
| rs4970834 [12-16] | 1 | 109814880 | CELSR2 | G | 0.231 | 0.340 | 100.00 | 0.76 | 0.78 | 0.49881 | 1.09 (0.85-1.40) |
| rs611917 [12, 13] | 1 | 109815252 | CELSR2 | G | 0.325 | 0.932 | 100.00 | 0.32 | 0.33 | 0.92247 | 1.01 (0.81-1.26) |
| rs12740374 [12, 13] | 1 | 109817590 | CELSR2 | C | 0.240 | 0.469 | 99.86 | 0.76 | 0.76 | 0.71271 | 1.05 (0.82-1.34) |
| rs660240 [12, 13] | 1 | 109817838 | CELSR2 | A | 0.232 | 0.595 | 98.86 | 0.23 | 0.23 | 0.87759 | 1.02 (0.80-1.31) |
| rs602633 [12, 13] | 1 | 109821511 | CELSR2 | A | 0.235 | 0.401 | 99.72 | 0.23 | 0.24 | 0.83040 | 1.03 (0.80-1.31) |
| rs2144300 [12] | 1 | 230294916 | GALNT2 | G | 0.350 | 0.619 | 99.72 | 0.34 | 0.36 | 0.56836 | 1.07 (0.86-1.33) |
| rs10198175 [12] | 2 | 21133883 | APOB | G | 0.080 | 0.610 | 100.00 | 0.92 | 0.93 | 0.48200 | 1.15 (0.78-1.69) |
| rs10495712 [12] | 2 | 21196112 | APOB | T | 0.242 | 0.257 | 99.86 | 0.24 | 0.25 | 0.65420 | 1.06 (0.83-1.35) |
| rs7557067 | 2 | 21208211 | APOB | T | 0.244 | 0.682 | 100.00 | 0.74 | 0.78 | 0.10331 | 1.22 (0.96-1.56) |
| rs673548 [12, 13] | 2 | 21237544 | APOB | G | 0.229 | 0.831 | 99.86 | 0.75 | 0.79 | 0.07327 | 1.26 (0.98-1.61) |
| rs1713222 [12, 13] | 2 | 21271323 | APOB | T | 0.110 | 0.846 | 99.86 | 0.10 | 0.12 | 0.29882 | 1.19 (0.85-1.67) |
| rs541041 [12] | 2 | 21294975 | APOB | C | 0.199 | 0.555 | 99.86 | 0.19 | 0.21 | 0.47184 | 1.10 (0.85-1.43) |
| rs1429974 [12] | 2 | 21300770 | APOB | A | Excluded |  |  |  |  |  |  |
| rs754524 [12] | 2 | 21311541 | APOB | C | 0.229 | 1.000 | 98.72 | 0.23 | 0.23 | 0.76721 | 1.04 (0.81-1.33) |
| rs312985 [12] | 2 | 21378805 | APOB | A | 0.204 | 0.488 | 99.86 | 0.19 | 0.21 | 0.35619 | 1.13 (0.87-1.46) |
| rs506585 [12-14] | 2 | 21397182 | APOB | C | 0.203 | 0.560 | 100.00 | 0.20 | 0.21 | 0.45350 | 1.10 (0.85-1.43) |
| rs949790 [12] | 2 | 21449987 | APOB | G | 0.241 | 0.918 | 100.00 | 0.75 | 0.77 | 0.28102 | 1.14 (0.90-1.46) |
| rs13398007 [12] | 2 | 21469336 | APOB | A | 0.131 | 1.000 | 100.00 | 0.12 | 0.14 | 0.27860 | 1.19 (0.87-1.62) |
| rs1260326 [12-16] | 2 | 27730940 | GCKR | T | 0.406 | 0.531 | 99.72 | 0.40 | 0.42 | 0.42688 | 1.09 (0.88-1.35) |
| rs780094 [12-16][29] | 2 | 27741237 | GCKR | T | 0.408 | 0.212 | 100.00 | 0.40 | 0.42 | 0.49165 | 1.08 (0.87-1.33) |
| rs1260333 [12] | 2 | 27748624 | GCKR | G | 0.459 | 0.058 | 100.00 | 0.54 | 0.55 | 0.68891 | 1.04 (0.85-1.29) |
| rs1919127 [12] | 2 | 27801493 | GCKR | C | 0.295 | 0.147 | 99.72 | 0.28 | 0.31 | 0.33066 | 1.12 (0.89-1.41) |
| rs7700965 [12] | 5 | 74356857 | HMGCR | T | 0.362 | 0.870 | 99.57 | 0.62 | 0.66 | 0.08778 | 1.21 (0.97-1.50) |
| rs3923323 [12] | 5 | 74372141 | HMGCR | A | 0.179 | 0.441 | 100.00 | 0.80 | 0.84 | 0.05531 | 1.31 (0.99-1.72) |
| rs4345300 [12] | 5 | 74376571 | HMGCR | C | 0.365 | 0.871 | 99.57 | 0.61 | 0.66 | 0.07468 | 1.22 (0.98-1.52) |
| **rs2035191 [7]** | **5** | **74413713** | **HMGCR** | **A** | **0.190** | **0.625** | **100.00** | **0.79** | **0.83** | **0.02867** | **1.35 (1.03-1.77)** |
| rs1422697 | 5 | 74445817 | HMGCR | G | 0.365 | 0.935 | 99.86 | 0.61 | 0.66 | 0.08999 | 1.21 (0.97-1.50) |
| **rs1551894 [7]** | **5** | **74570531** | **HMGCR** | **C** | **0.233** | **0.673** | **99.86** | **0.74** | **0.79** | **0.02670** | **1.32 (1.03-1.70)** |
| rs2335418 [7] | 5 | 74603479 | HMGCR | C | 0.434 | 0.591 | 99.57 | 0.55 | 0.59 | 0.11478 | 1.19 (0.96-1.46) |
| rs10474433 [7] | 5 | 74616843 | HMGCR | A | 0.361 | 0.624 | 100.00 | 0.62 | 0.66 | 0.10729 | 1.20 (0.96-1.49) |
| rs3761740 [7] | 5 | 74632133 | HMGCR | C | 0.119 | 0.030 | 99.57 | 0.88 | 0.89 | 0.59339 | 1.09 (0.79-1.51) |
| **rs3846662 [7, 12, 22]** | **5** | **74651084** | **HMGCR** | **T** | **0.442** | **0.702** | **99.57** | **0.53** | **0.59** | **0.03609** | **1.25 (1.01-1.55)** |
| rs4385188 [7] | 5 | 74734362 | HMGCR | A | 0.235 | 0.346 | 99.86 | 0.75 | 0.79 | 0.06716 | 1.26 (0.98-1.61) |
| rs6872314 [7] | 5 | 74736065 | HMGCR | A | 0.240 | 0.215 | 99.57 | 0.74 | 0.78 | 0.09340 | 1.23 (0.97-1.58) |
| rs6896136 [7] | 5 | 74787310 | HMGCR | T | 0.413 | 0.484 | 99.86 | 0.56 | 0.61 | 0.05583 | 1.23 (0.99-1.52) |
| rs1051795 [7] | 5 | 74896798 | HMGCR | C | 0.220 | 0.442 | 99.72 | 0.76 | 0.80 | 0.07164 | 1.26 (0.98-1.63) |
| rs904743 [7] | 5 | 74917862 | HMGCR | T | 0.117 | 0.140 | 99.86 | 0.87 | 0.90 | 0.06707 | 1.36 (0.98-1.89) |
| rs34358 [7] | 5 | 74965122 | HMGCR | T | 0.378 | 0.472 | 99.86 | 0.61 | 0.64 | 0.20317 | 1.15 (0.93-1.43) |
| rs2240466 [7] | 7 | 72856269 | MLXIPL | C | 0.100 | 0.096 | 100.00 | 0.89 | 0.91 | 0.08500 | 1.36 (0.96-1.94) |
| rs1178979 [30] | 7 | 72856430 | MLXIPL | T | 0.151 | 0.144 | 100.00 | 0.83 | 0.87 | 0.05728 | 1.33 (0.99-1.78) |
| rs714052 [7, 24] | 7 | 72864869 | MLXIPL | A | 0.100 | 0.096 | 100.00 | 0.89 | 0.91 | 0.08500 | 1.36 (0.96-1.94) |
| rs17145738 [11, 15, 27] | 7 | 72982874 | MLXIPL | G | 0.102 | 0.063 | 100.00 | 0.88 | 0.91 | 0.07384 | 1.37 (0.97-1.95) |
| rs2286276 [7] | 7 | 72987354 | MLXIPL | C | 0.220 | 0.827 | 100.00 | 0.76 | 0.80 | 0.12142 | 1.22 (0.95-1.57) |
| **rs11974409 [7, 12]** | **7** | **72989390** | **MLXIPL** | **T** | **0.154** | **0.112** | **99.86** | **0.83** | **0.87** | **0.03326** | **1.37 (1.02-1.84)** |
| rs264 [7, 23] | 8 | 19813180 | LPL | G | 0.112 | 0.086 | 99.86 | 0.88 | 0.90 | 0.18394 | 1.25 (0.90-1.75) |
| **rs328 [12, 15, 23, 25, 27, 29]** | **8** | **19819724** | **LPL** | **G** | **0.057** | **1.000** | **100.00** | **0.93** | **0.96** | **0.02156** | **1.72 (1.08-2.75)** |
| **rs12679834 [23]** | **8** | **19820433** | **LPL** | **A** | **0.058** | **1.000** | **100.00** | **0.93** | **0.96** | **0.01657** | **1.76 (1.10-2.80)** |
| rs2197089 [11] | 8 | 19826373 | LPL | G | 0.434 | 0.282 | 99.57 | 0.42 | 0.45 | 0.16920 | 1.16 (0.94-1.43) |
| rs10105606 [30] | 8 | 19827848 | LPL | G | 0.290 | 0.201 | 99.86 | 0.69 | 0.73 | 0.07652 | 1.23 (0.98-1.55) |
| **rs10096633 [7, 23, 27]** | **8** | **19830921** | **LPL** | **G** | **0.072** | **1.000** | **100.00** | **0.91** | **0.94** | **0.02056** | **1.63 (1.07-2.47)** |
| **rs1919484 [17, 23]** | **8** | **19869676** | **LPL** | **C** | **0.256** | **0.373** | **100.00** | **0.72** | **0.77** | **0.03347** | **1.30 (1.02-1.65)** |
| **rs2410630 [7]** | **8** | **19875100** | **LPL** | **C** | **0.396** | **0.059** | **99.86** | **0.57** | **0.64** | **0.01758** | **1.30 (1.05-1.61)** |
| rs7016880 [23] | 8 | 19876746 | LPL | G | 0.055 | 1.000 | 100.00 | 0.93 | 0.96 | 0.06214 | 1.56 (0.97-2.49) |
| rs6993414 [11, 23] | 8 | 19902918 | LPL | A | 0.063 | 0.748 | 100.00 | 0.93 | 0.95 | 0.08281 | 1.47 (0.95-2.29) |
| rs6586891 [11] | 8 | 19914598 | LPL | C | 0.356 | 0.071 | 100.00 | 0.34 | 0.37 | 0.27000 | 1.13 (0.91-1.41) |
| rs17321515 [11, 15, 27] | 8 | 126486409 | TRIB1 | T | 0.476 | 0.326 | 99.57 | 0.45 | 0.50 | 0.06058 | 1.22 (0.99-1.51) |
| rs2954029 [11, 24, 30] | 8 | 126490972 | TRIB1 | A | 0.482 | 0.114 | 100.00 | 0.46 | 0.51 | 0.08426 | 1.20 (0.98-1.48) |
| rs6987702 [7] | 8 | 126504726 | TRIB1 | A | 0.270 | 0.180 | 99.57 | 0.73 | 0.73 | 0.72900 | 1.04 (0.82-1.32) |
| rs471364 [24] | 9 | 15289578 | TTC39B | C | 0.100 | 0.096 | 100.00 | 0.10 | 0.10 | 0.55749 | 1.11 (0.78-1.57) |
| rs3905000 [7] | 9 | 107657070 | ABCA1 | C | 0.108 | 0.555 | 100.00 | 0.89 | 0.90 | 0.72380 | 1.06 (0.76-1.49) |
| rs1883025 [24] | 9 | 107664301 | ABCA1 | A | 0.210 | 0.426 | 99.72 | 0.20 | 0.23 | 0.17779 | 1.19 (0.92-1.54) |
| rs174547 [24] | 11 | 61570783 | FADS2 | T | 0.357 | 0.284 | 99.57 | 0.64 | 0.65 | 0.86616 | 1.02 (0.82-1.27) |
| rs174556 [27] | 11 | 61580635 | FADS2 | A | 0.311 | 0.160 | 99.72 | 0.31 | 0.32 | 0.72050 | 1.04 (0.83-1.31) |
| rs174566 | 11 | 61592362 | FADS2 | G | 0.352 | 0.323 | 99.86 | 0.35 | 0.36 | 0.78132 | 1.03 (0.83-1.28) |
| rs174570 [7] | 11 | 61597212 | FADS2 | T | 0.183 | 0.528 | 100.00 | 0.18 | 0.19 | 0.70062 | 1.05 (0.80-1.38) |
| rs2851682 [7] | 11 | 61616012 | FADS2 | T | 0.154 | 0.666 | 100.00 | 0.84 | 0.85 | 0.68730 | 1.06 (0.79-1.42) |
| rs480878 [7] | 11 | 116522854 | APOA1 | A | 0.119 | 0.074 | 100.00 | 0.11 | 0.13 | 0.31194 | 1.18 (0.86-1.63) |
| rs4938303 [30] | 11 | 116584987 | APOA5 | G | 0.352 | 0.741 | 100.00 | 0.33 | 0.37 | 0.07714 | 1.22 (0.98-1.52) |
| **rs2000571 [11]** | **11** | **116585533** | **APOA1** | **A** | **0.267** | **0.847** | **99.72** | **0.24** | **0.29** | **0.04296** | **1.28 (1.01-1.62)** |
| rs12272004 [7] | 11 | 116603724 | APOA1 | C | 0.088 | 0.477 | 100.00 | 0.91 | 0.92 | 0.64960 | 1.09 (0.75-1.58) |
| rs2892 [15, 27] | 11 | 116619073 | APOA1 | G | 0.087 | 0.637 | 100.00 | 0.91 | 0.92 | 0.78487 | 1.05 (0.73-1.53) |
| rs12292921 [7] | 11 | 116621963 | APOA1 | T | 0.085 | 0.627 | 100.00 | 0.91 | 0.92 | 0.63773 | 1.09 (0.75-1.59) |
| rs964184 [11, 24, 30] | 11 | 116648917 | APOA5 | C | 0.149 | 0.657 | 99.86 | 0.13 | 0.16 | 0.11467 | 1.27 (0.94-1.70) |
| **rs6589566 [10]** | **11** | **116652423** | **APOA5** | **C** | **0.067** | **0.761** | **100.00** | **0.05** | **0.08** | **0.02932** | **1.60 (1.05-2.45)** |
| rs35120633 [7] | 11 | 116655600 | APOA1 | G | 0.079 | 1.000 | 100.00 | 0.92 | 0.92 | 0.68054 | 1.08 (0.74-1.60) |
| rs3135506 [7, 12] | 11 | 116662407 | APOA1 | C | 0.081 | 1.000 | 99.86 | 0.92 | 0.92 | 0.60835 | 1.11 (0.75-1.62) |
| **rs662799 [11, 12]** | **11** | **116663707** | **APOA1** | **C** | **0.063** | **0.511** | **100.00** | **0.05** | **0.08** | **0.01427** | **1.73 (1.11-2.69)** |
| rs2075292 [7, 25] | 11 | 116732512 | APOA1 | G | 0.142 | 1.000 | 100.00 | 0.13 | 0.16 | 0.14440 | 1.25 (0.93-1.69) |
| rs588918 [7] | 11 | 116856642 | APOA1 | T | 0.135 | 0.873 | 100.00 | 0.12 | 0.15 | 0.05243 | 1.35 (1.00-1.84) |
| rs1351452 [7] | 11 | 116943354 | APOA1 | T | 0.137 | 1.000 | 99.86 | 0.12 | 0.15 | 0.07160 | 1.32 (0.98-1.8) |
| rs10892151 [26] | 11 | 117531731 | APOA1 | A | 0.028 | 1.000 | 99.86 | 0.03 | 0.03 | 0.99299 | 1.00 (0.53-1.90) |
| rs2454722 | 12 | 123171218 | KNTC1/NIACR1 | C | 0.186 | 0.804 | 99.86 | 0.18 | 0.19 | 0.43769 | 1.11 (0.85-1.46) |
| rs601339 | 12 | 123174743 | NIACR1/KNTC1 | G | 0.179 | 0.798 | 99.72 | 0.18 | 0.18 | 0.70488 | 1.05 (0.80-1.39) |
| rs1798219 | 12 | 123180061 | GPR109A/B | G | 0.185 | 0.617 | 100.00 | 0.18 | 0.19 | 0.55242 | 1.09 (0.83-1.42) |
| rs7972971 | 12 | 123182332 | KNTC1/NIACR1 | C | 0.424 | 0.075 | 99.43 | 0.41 | 0.44 | 0.29713 | 1.12 (0.91-1.38) |
| **rs7314976** | **12** | **123186900** | **NIACR1** | **A** | **0.209** | **0.301** | **98.58** | **0.19** | **0.23** | **0.03935** | **1.31 (1.01-1.70)** |
| rs676823 | 12 | 123187239 | NIACR1 | T | Excluded |  |  |  |  |  |  |
| rs676404 | 12 | 123187305 | NIACR1 | G | Excluded |  |  |  |  |  |  |
| rs17884481 | 12 | 123200693 | NIACR2 | G | 0.396 | 1.000 | 100.00 | 0.59 | 0.62 | 0.17707 | 1.16 (0.94-1.44) |
| rs1798192 | 12 | 123200768 | NIACR2 | G | 0.395 | 0.937 | 99.57 | 0.59 | 0.62 | 0.17214 | 1.16 (0.94-1.44) |
| rs2256572 | 12 | 123201508 | NIACR2/GPR81 | C | 0.399 | 0.937 | 99.86 | 0.59 | 0.62 | 0.23415 | 1.14 (0.92-1.41) |
| rs3922628 | 12 | 123209295 | NIACR2/GPR81 | A | Excluded |  |  |  |  |  |  |
| rs4775041 [7, 11, 27] | 15 | 58674695 | LIPC | G | 0.281 | 0.015 | 99.43 | 0.70 | 0.74 | 0.05156 | 1.26 (1.00-1.59) |
| rs166358 [7] | 15 | 58680805 | LIPC | G | 0.162 | 0.211 | 100.00 | 0.83 | 0.85 | 0.24192 | 1.19 (0.89-1.58) |
| rs2043085 [7] | 15 | 58680954 | LIPC | G | 0.327 | 0.087 | 99.57 | 0.65 | 0.69 | 0.11261 | 1.20 (0.96-1.50) |
| rs1532085 [7, 12, 27] | 15 | 58683366 | LIPC | C | 0.328 | 0.086 | 99.57 | 0.65 | 0.70 | 0.05638 | 1.24 (0.99-1.55) |
| rs415799 [7] | 15 | 58690754 | LIPC | A | 0.464 | 0.705 | 99.86 | 0.52 | 0.55 | 0.25660 | 1.13 (0.92-1.39) |
| rs397923 [7] | 15 | 58692118 | LIPC | T | 0.378 | 0.109 | 100.00 | 0.61 | 0.64 | 0.17976 | 1.16 (0.93-1.44) |
| rs487766 [7] | 15 | 58693860 | LIPC | A | 0.169 | 0.501 | 99.43 | 0.82 | 0.85 | 0.12390 | 1.25 (0.94-1.65) |
| rs11856159 [7] | 15 | 58699010 | LIPC | C | 0.156 | 0.886 | 100.00 | 0.83 | 0.86 | 0.11544 | 1.26 (0.94-1.69) |
| rs573922 | 15 | 58740094 | LIPC | A | 0.190 | 0.807 | 100.00 | 0.81 | 0.81 | 0.72666 | 1.05 (0.80-1.37) |
| rs2241770 [7] | 16 | 56866196 | CETP | A | 0.086 | 1.000 | 99.86 | 0.91 | 0.92 | 0.44249 | 1.16 (0.80-1.68) |
| rs16962767 [7] | 16 | 56873789 | CETP | T | 0.085 | 1.000 | 100.00 | 0.91 | 0.92 | 0.44568 | 1.16 (0.79-1.69) |
| **rs12448528 [7, 23]** | **16** | **56985555** | **CETP** | **A** | **0.196** | **0.187** | **99.72** | **0.17** | **0.22** | **0.01530** | **1.39 (1.06-1.81)** |
| **rs173539 [23, 24]** | **16** | **56988044** | **CETP** | **C** | **0.343** | **1.000** | **99.86** | **0.59** | **0.73** | **1.73E-07** | **1.81 (1.45-2.27)** |
| **rs12708967 [23]** | **16** | **56993211** | **CETP** | **C** | **0.166** | **0.277** | **99.86** | **0.14** | **0.19** | **0.01132** | **1.44 (1.08-1.91)** |
| **rs3764261 [7, 11, 12, 14, 23, 27]** | **16** | **56993324** | **CETP** | **G** | **0.339** | **0.801** | **99.86** | **0.60** | **0.73** | **1.14E-07** | **1.83 (1.46-2.29)** |
| **rs1800775 [7, 15, 17, 18, 23, 27, 29]** | **16** | **56995236** | **CETP** | **C** | **0.446** | **0.012** | **99.57** | **0.37** | **0.52** | **1.32E-08** | **1.85 (1.50-2.29)** |
| **rs711752 [23, 25]** | **16** | **56996211** | **CETP** | **C** | **0.462** | **0.324** | **99.72** | **0.47** | **0.61** | **4.17E-07** | **1.73 (1.40-2.13)** |
| **rs708272 [18]** | **16** | **56996288** | **CETP** | **G** | **0.461** | **0.197** | **99.72** | **0.47** | **0.61** | **4.27E-07** | **1.73 (1.40-2.13)** |
| **rs1864163 [7, 11, 18, 23]** | **16** | **56997233** | **CETP** | **A** | **0.246** | **0.542** | **100.00** | **0.21** | **0.29** | **0.00055** | **1.54 (1.20-1.96)** |
| **rs9929488 [23]** | **16** | **56998572** | **CETP** | **G** | **0.272** | **0.569** | **99.86** | **0.23** | **0.32** | **4.31E-05** | **1.64 (1.29-2.08)** |
| **rs7203984 [7, 23]** | **16** | **56999258** | **CETP** | **G** | **0.193** | **0.717** | **99.86** | **0.15** | **0.24** | **3.63E-06** | **1.88 (1.44-2.47)** |
| **rs9939224 [23]** | **16** | **57002732** | **CETP** | **T** | **0.188** | **0.324** | **99.86** | **0.14** | **0.24** | **2.69E-07** | **2.04 (1.55-2.69)** |
| **rs7205804 [7, 23, 25]** | **16** | **57004889** | **CETP** | **C** | **0.459** | **0.173** | **100.00** | **0.48** | **0.60** | **4.80E-06** | **1.64 (1.32-2.02)** |
| **rs11076175 [23]** | **16** | **57006378** | **CETP** | **G** | **0.173** | **0.432** | **100.00** | **0.13** | **0.22** | **1.17E-05** | **1.87 (1.41-2.48)** |
| **rs289714 [23]** | **16** | **57007451** | **CETP** | **G** | **0.144** | **0.540** | **99.15** | **0.12** | **0.17** | **0.00312** | **1.57 (1.16-2.13)** |
| **rs5880 [18, 25]** | **16** | **57015091** | **CETP** | **G** | **0.028** | **0.094** | **100.00** | **0.02** | **0.04** | **0.02298** | **2.15 (1.09-4.22)** |
| **rs1800777 [18, 25]** | **16** | **57017319** | **CETP** | **T** | **0.020** | **1.000** | **100.00** | **0.01** | **0.03** | **0.04073** | **2.26 (1.01-5.02)** |
| rs16965220 [30] | 16 | 57065121 | CETP | C | 0.284 | 0.853 | 100.00 | 0.71 | 0.72 | 0.48648 | 1.09 (0.86-1.37) |
| **rs2271293 [24]** | **16** | **67902070** | **LCAT** | **G** | **0.158** | **0.255** | **100.00** | **0.82** | **0.87** | **0.01204** | **1.45 (1.08-1.94)** |
| **rs255049 [27]** | **16** | **68013471** | **LCAT** | **A** | **0.205** | **0.084** | **100.00** | **0.77** | **0.82** | **0.01875** | **1.37 (1.05-1.77)** |
| **rs9891572 [27]** | **17** | **2428508** |  | **C** | **0.207** | **0.820** | **100.00** | **0.76** | **0.83** | **0.00221** | **1.50 (1.16-1.95)** |
| **rs6507945 [17]** | **18** | **47243912** | **LIPG** | **A** | **0.455** | **0.447** | **99.57** | **0.42** | **0.49** | **0.01510** | **1.30 (1.05-1.6)** |
| rs6511720 [7, 11, 12, 15, 24, 27] | 19 | 11202306 | LDLR | G | 0.099 | 0.670 | 99.72 | 0.89 | 0.92 | 0.08233 | 1.37 (0.96-1.95) |
| rs2228671 [7, 28] | 19 | 11210912 | LDLR | C | 0.112 | 1.000 | 100.00 | 0.87 | 0.90 | 0.07550 | 1.35 (0.97-1.90) |
| rs16996148 [11, 15, 27] | 19 | 19658472 | CILP2 | G | 0.079 | 0.188 | 99.72 | 0.92 | 0.92 | 0.83125 | 1.04 (0.71-1.54) |
| rs17216525 [24] | 19 | 19662220 | CILP2 | G | 0.078 | 0.600 | 99.57 | 0.92 | 0.92 | 0.79014 | 1.05 (0.71-1.56) |
| rs12610185 [7] | 19 | 19721722 | NCAN | A | 0.080 | 0.193 | 99.86 | 0.08 | 0.08 | 0.94436 | 1.01 (0.69-1.49) |
| rs2304128 [7] | 19 | 19746151 | NCAN | G | 0.077 | 0.593 | 100.00 | 0.92 | 0.92 | 0.90125 | 1.03 (0.69-1.52) |
| rs2304130 [7, 30] | 19 | 19789528 | CILP2 | C | 0.059 | 0.728 | 100.00 | 0.05 | 0.06 | 0.42016 | 1.20 (0.77-1.87) |
| rs2965101 [7] | 19 | 45237812 | TOMM40 | T | 0.301 | 0.591 | 99.86 | 0.69 | 0.70 | 0.69370 | 1.05 (0.83-1.32) |
| rs4803750 [7, 12, 28] | 19 | 45247627 | TOMM40 | T | 0.084 | 1.000 | 99.86 | 0.91 | 0.92 | 0.49173 | 1.14 (0.78-1.67) |
| rs1871045 [7] | 19 | 45326768 | TOMM40 | C | 0.323 | 0.931 | 99.86 | 0.66 | 0.69 | 0.31173 | 1.12 (0.90-1.40) |
| rs6859 [7] | 19 | 45382034 | TOMM40 | A | 0.425 | 0.356 | 99.86 | 0.42 | 0.43 | 0.89436 | 1.01 (0.82-1.25) |
| rs157580 [7, 27] | 19 | 45395266 | APOE | C | 0.326 | 0.797 | 99.72 | 0.32 | 0.33 | 0.86218 | 1.02 (0.82-1.28) |
| rs2075650 [7, 27, 28] | 19 | 45395619 | APOE | G | 0.159 | 0.066 | 99.86 | 0.15 | 0.17 | 0.55712 | 1.09 (0.82-1.45) |
| **rs405509 [7]** | **19** | **45408836** | **TOMM40** | **A** | **0.434** | **0.443** | **100.00** | **0.41** | **0.46** | **0.03776** | **1.25 (1.01-1.54)** |
| rs7679 [24] | 20 | 44576502 | PLTP | G | 0.175 | 1.000 | 99.86 | 0.16 | 0.19 | 0.08349 | 1.28 (0.97-1.68) |

*^*SNPs significantly associated with HDL cholesterol level are marked in bold^*
